# Video Transmission for Third Generation Wireless Communication Systems

**DOI:** 10.6028/jres.106.020

**Published:** 2001-04-01

**Authors:** H. Gharavi, S. M. Alamouti

**Affiliations:** National Institute of Standards and Technology, Gaithersburg, MD 20899-8920; Cadence Design Systems Inc, San Jose, CA 95134

**Keywords:** multimedia communications, third generation mobile systems, WCDMA, wireless video

## Abstract

This paper presents a twin-class unequal protected video transmission system over wireless channels. Video partitioning based on a separation of the Variable Length Coded (VLC) Discrete Cosine Transform (DCT) coefficients within each block is considered for constant bitrate transmission (CBR). In the splitting process the fraction of bits assigned to each of the two partitions is adjusted according to the requirements of the unequal error protection scheme employed. Subsequently, partitioning is applied to the ITU-T H.263 coding standard. As a transport vehicle, we have considered one of the leading third generation cellular radio standards known as WCDMA. A dual-priority transmission system is then invoked on the WCDMA system where the video data, after being broken into two streams, is unequally protected. We use a very simple error correction coding scheme for illustration and then propose more sophisticated forms of unequal protection of the digitized video signals. We show that this strategy results in a significantly higher quality of the reconstructed video data when it is transmitted over time-varying multipath fading channels.

## 1. Introduction

The wireless revolution in the 1980s was primarily driven by market demands for mobile radio voice communications. The first generation analog wireless communication systems used old radio technologies combined with novel cellular network planning [1] to provide transparent ubiquitous mobile access to users. Initially, the cellular market was a niche market targeted at business people. As the price of the user terminals and services dropped due to an unprecedented exponential increase in demand and related economies of scale, cellular radios became the communications tool of choice for the masses. Today, there are in the neighborhood of 300 million mobile phone users in the world. It is expected that in the next decade the number of users will reach the one billion mark.

The second generation wireless systems were introduced in the 1990s and were primarily an evolutionary step towards improving the capacity of cellular systems through digitization of voice and efficient digital modulation schemes. These systems also provided additional features such as security, short messaging, and circuit-switched data.

We are now in the midst of another evolution planned for deployment in the 2000s. This evolution is once again driven by the need for greater bandwidth in anticipation of further demand for voice services. However, the new wireless standards will also provide a pipeline for broadband services such as enhanced high rate data and multimedia services. The global growth of interest in the Internet and in digitized audio and video, and the demand for such services through the fixed communications networks, is an important factor. Although there are currently no applications with great mass market appeal that require broadband wireless access, it is anticipated that the popularity of these services in fixed networks will eventually impact the market for wireless communications.

The next generation wireless systems are required to have voice services of wireline quality and to provide high bit rate data services of 144 kbit/s to 2 Mbit/s depending on the radio environment. At the same time, they are to operate reliably in different types of environments: macro, micro, and pico cellular; urban, suburban, and rural; indoor and outdoor. In other words, the next generation systems are supposed to have better quality and coverage, be more power and bandwidth efficient, and be deployed in diverse environments. These high data rates make video transmission possible for a number of applications such as video conferencing, emergency medical services, and site surveys. However, since most existing video compression standards [7,8,9,10,11] have been developed for relatively benign, nearly error-free environments, they cannot be directly applied in a hostile mobile domain. This is mainly due to the extensive employment of variable length coding techniques, which are efficient in bitrate reduction terms, but are error-sensitive. A single transmission error may result in an undecodable string of bits. One effective method of protecting the compressed video signal is to split the coded video signal into a number of separate bitstreams where each can be transmitted via a separate channel having a different degree of error protection [12,13,14]. The bitstream splitting can be accomplished by taking into consideration the perceptual significance of coded video, where better protection is provided for the transmission of the perceptually more important bits. In this paper, such a strategy has been considered for one of the leading third generation cellular radio standards known as WCDMA.

The paper begins with an overview of the WCDMA radio standard [2]. Then, a video partitioning scheme based on the ITU-T H.263 standard, is presented. This is followed by a very simple error protection coding scheme for the transmission of partitioned video over IMT-2000 channels. The transmission system model and its parameters are then discussed. This is followed by simulation results that evaluate the transmission of partitioned video using the downlink WCDMA physical layer as a transport vehicle. Finally, for possible future investigations, more sophisticated forms of unequal protection for the digitized video signals are discussed.

## 2. Review of WCDMA Radio Standards

It seems that a common international standard for the third generation wireless communications systems may soon be adopted. The international standard may have three radio access schemes and is currently being harmonized by the Third Generation Partnership Project (3GPP). The three radio access schemes are the Frequency-Division-Duplex (FDD) mode, the Time-Division Duplex (TDD) mode and the Multi-Carrier (MC) mode. The FDD mode is a direct sequence (DS) CDMA scheme initially proposed by Japan’s Association of Radio Industries and Businesses (ARIB) and the European Telecommunications Standards Institute (ETSI). The original proposed radio standard was called the UMTS Terrestrial Radio Access or UTRA which included a TDD mode for deployment in unpaired bands. The multi-carrier mode is based on the Telecommunications Industry Association (TIA) standard known as CDMA2000 or IS2000.

In the next section, we discuss the physical layer parameters and spreading and modulation for the FDD mode of 3GPP radio access scheme and we will refer to it as WCDMA [2]. Please note that the information in this paper may not be consistent with the latest developments in the standard. We intend to give the reader a basic understanding of concepts and do not intend this paper to be a replacement for the detailed specifications, which may not be finalized. We then simulate the proposed video coding scheme over the downlink physical layer of WCDMA and report the performance results.

### 2.1 The WCDMA Airlink Parameters

The modulation chip rate for WCDMA is 3.84 Mega Chips Per Second (Mcps)^1^. The number of chips per modulation symbol is called the Spreading Factor (SF). The specified pulse shaping roll off factor is 0.22. This results in an effective minimum bandwidth of 3.84(1+0.22) which is less than 5 MHz. The nominal channel spacing for WCDMA 5 MHz. The WCDMA carriers are specified on a grid with a resolution of 200 kHz. Although the typical separation between the carriers is 5 MHz, it may vary depending on the deployment scenario. The frame length is fixed at 10 ms, which corresponds to 38 400 chips. The number of bits or symbols in a frame may vary depending on the data rate, which is variable. The variable data rates are supported using variable spreading factors.

The transmissions from all the physical channels within a 5 MHz band are code division multiplexed and transmitted over the same band. We discuss two channels in this paper: i) Dedicated Physical Data Channels (DPDCH) and, ii) Dedicated Physical Control Channels (DPCCH). Table 1 summarizes the bit rates, symbol rates, and the spreading factors for these channels. All channels are spread by a spreading code with a spreading factor (SF) that may vary from 4 to 512 on the downlink and 4 to 256 on the uplink, depending on the data rate of the channel. The spreading factors must be an integer power of 2.

### 2.2 The WCDMA Downlink

**Fig. 1:**
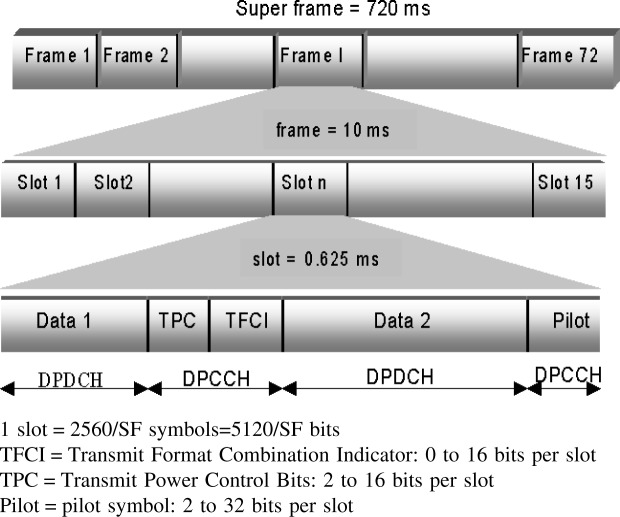
The frame structure definitions for WCDMA downlink DPDCH/DPCCH.

The frame structure for the downlink DPCCH/DPDCH channel is shown in Fig. 1. A super frame is made up of 72, 10 ms frames. Each frame has 15 equal-length slots. As shown in Fig. 1, the DPCCH and DPDCH are time multiplexed within the same slot.

The DPCCH channel consists of pilot symbols, transmit power-control (TPC) bits and transport format combination indicator (TFCI) bits (please note that TFCI is optional and is not used for fixed rate services). The DPDCH channels contain bearer data. The number of bits transmitted in a single slot depends on the data rate of the channel.

### 2.3 The Downlink Traffic Channel (Forward DPDCH/DPCCH)

**Fig. 2:**
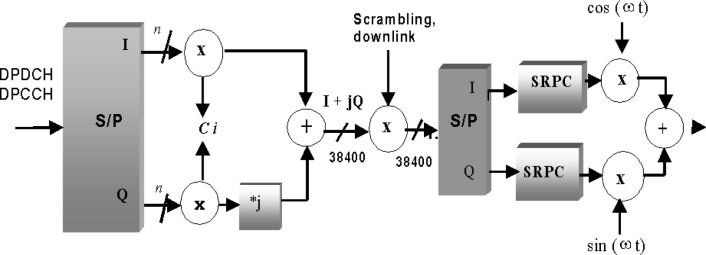
The transmission process for DPCCH/DPDCH channels in a 10 ms frame.

Figure 2 shows the downlink DPCCH/DPDCH transmit processing for one channel in a 10 ms frame. The process shown in the figure is repeated every 10 ms^2^. A DPCCH/DPDCH channel carries 2*n* bits of information in every 10 ms frame. The actual number of bits depends on the data rate and hence the spreading factor. The 2*n* bits are mapped onto n QPSK complex symbols. The QPSK (quadrature Phase Shift Keying) symbols (in the *i*th channel) are first spread using a spreading code, *C_i_* of length *L*, to produce 38 400 chips. In other words, *nL* = 38 400. The resulting chips on the I and Q channels are then treated as a single complex value and are chip-wise scrambled by a complex long code *S*_scramb_ of length 38 400. The resulting chips are then weighted and combined with chips from all the other downlink channels. The complex valued chips are then separated into I and Q symbols and are passed through square root raised cosine filters (SRRC) with roll-off factor *a* = 0.22. The I-channel and Q-channel signals are then RF converted using quadrature (cos *ωt* and sin *ωt*) carriers, and finally transmitted through the air. The spreading codes are orthogonal sequences whose elements are either 1 or −1, and so are the elements of the non-orthogonal long scrambling codes.

### 2.4 The WCDMA Uplink

**Fig. 3:**
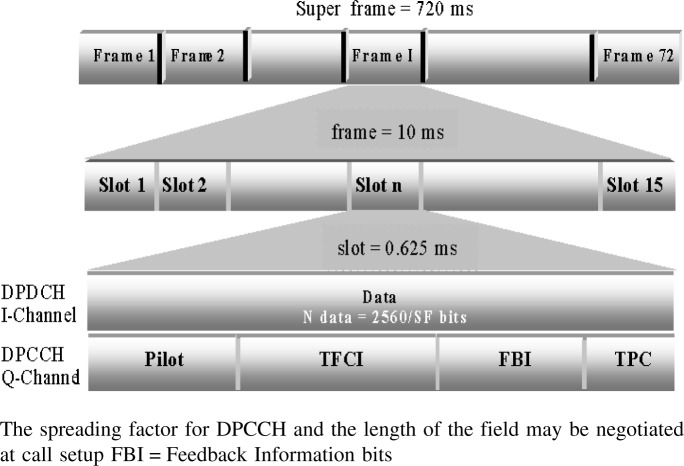
The frame structure definitions for WCDMA uplink DPDCH/DPCCH.

**Fig. 4:**
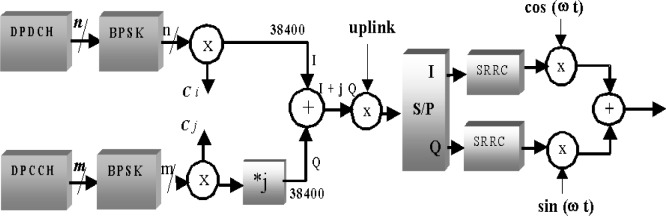
Transmit processing for DPCCH/DPDCH uplink channels.

The frame structure for the uplink DPCCH/DPDCH channel is shown in Fig. 3. The basic parameters for uplink framing are the same as the downlink. The only difference is that the DPDCH and DPCCH are not time multiplexed. Figure 4 shows the uplink DPCCH/DPDCH transmit processing for one channel. Unlike the forward channel, the DPDCH and DPCCH are transmitted in parallel and are separated using different channelization (spreading) codes. Also, the reverse transmissions carry feedback information bits (FBI).

The *n* information bits from the DPDCH channel are BPSK modulated resulting in *n* symbols which are then spread using a spreading code *C_i_* of length *L*_d_ to produce 38 400 chips. Similarly, m DPCCH bits are mapped onto m BPSK (Binary Phase Shift Keying) symbols which are spread using a spreading code *C_j_* of length *L*_c_ to produce 38 400 chips. In other words, *n*L_d_ = *mL*_c_ = 38 400. The chips from the DPDCH are mapped onto the in-phase axis and those from DPCCH are mapped on to the quadrature axis of the signal constellation. This is shown in Fig. 4 by a *j* multiply followed by an add operation. The signal constellation is, in effect, a QPSK constellation. Therefore, the modulation scheme for the reverse channel is called dual-channel QPSK. The resulting chips are then scrambled by a complex scrambling code *S*_uplink_. The uplink scrambling code is either a complex short code which is 256 chips long or a complex long code which is 38 400 chips long. When the short code is used, the code is repeated 150 times in order to scramble the 38 400 chip frame. The resulting chips are then passed through a square root raised cosine filter (SRRC) with roll-off factor *a* = 0.22, are IF/RF converted and then transmitted through the air.

### 2.5 The Spreading and Scrambling Codes

There are two major types of codes specified for WCDMA channels: orthogonal spreading codes and non-orthogonal long and short scrambling codes. The orthogonal spreading codes are used for channelization and the short and long scrambling codes are used for reducing inter-cell and intra-cell interference. In this paper, we do not discuss the detailed specifications of these codes. Instead, we will give you an overview of the different families of codes and their functions.

#### 2.5.1 Orthogonal Variable Spreading Factor (OVSF) Codes

These spreading codes, also known as channelization codes, are used to ensure orthogonality between the channels with different spreading factors and rates and are hence called Orthogonal Variable Spreading Factor (OVSF) codes [4]. On the downlink, the orthogonal spreading codes are used to separate the transmissions of the various traffic and control channels within a given cell. On the uplink, the use of spreading codes is not coordinated by the system. The base station and the mobile station agree only on the number of orthogonal channels and hence the number of codes, but the spreading codes used by the mobile stations is not negotiated. Therefore, two mobile stations in a given cell may use the same spreading code on the uplink. In such cases, the resulting interference is suppressed by the uplink scrambling codes which are unique to every mobile station in a given cell.

**Fig. 5:**
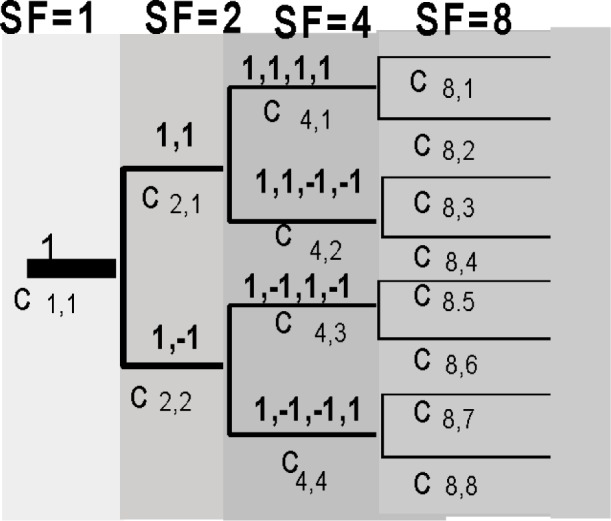
The code tree for the OVSF codes.

The OVSF codes are generated using the code tree shown in Fig. 5. The construction of these codes is very similar to Hadamard codes. Two branches emanate from each code in the code tree. The top emanating branch is simply the code from the mother branch repeated twice and the bottom branch is that same code followed by its negation. Not all the OVSF codes are mutually orthogonal. A given code can be used in a cell if and only if there are no other codes used on the path from that given code, to the root of the tree, or any code belonging to the sub-tree generated from that specific code. For instance, if C_4,1_ is used, then C_2,1_ and C_1,1_ may not be used in the same cell as they are in the path to the root. Also, C_8,1_ and C_8,2_ and all other codes derived from C_4,1_ cannot be used in the same cell. Depending on the required data rate, the length of the spreading code applied on a given DPCCH/DPDCH channel may vary from 4 to 512 on the downlink and 4 to 256 on the uplink (see Table 1).

#### 2.5.2 The Non-Orthogonal Scrambling Codes

There are three classes of scrambling codes used in WCDMA; 1) downlink long scrambling codes, 2) uplink short scrambling codes, 3) uplink long scrambling codes. In order to reduce the interference from the channels in adjacent cells in the downlink, every base station uses a long scrambling code. The downlink long scrambling codes are from the well-known family of Gold codes [5] built by the product of two maximum linear PN (Pseudo Noise) sequences. These codes have good cross-correlation properties and are widely used in commercial spread spectrum systems. The long code used on the forward DCDPCH/DCCPCH is 38 400 chips of a 2^15^−1 Gold Code.

The uplink short and long scrambling codes are used to suppress intercell and intracell interference from the various mobile stations at the base station receiver. The mobile stations in a given cell are each assigned a unique short scrambling code. These short scrambling codes are from the periodic extended S(2) code family of length 256. Because of the large number of these codes, it is possible to have a unique code for each mobile station in a given cell and also to make sure that the mobile stations in the neighboring cells are assigned different codes. The long code used on the reverse DCDPCH/DCCPCH is 38 400 chips of a complex 2^25^−1 Gold Code. The quadrature component of the code is a decimated and shifted version of the in-phase component of the same Gold code. The quadrature component is also multiplied by repetitions of a 1 and −1 sequence to improve the envelope properties of the signal. The WCDMA has provisions for multi-user detection where a base station can detect multiple users at the same time, hence, increasing the effective signal-to-noise-ratio (SNR) of all the users. Only short scrambling codes are applied when multi-user detection is used at the base station receiver. Otherwise, only long scrambling codes are used.

## 3. Source Coding and Video Partitioning

Given a certain total bitrate budget, use of strong error-correction coding increases the error resilience of the video transceiver scheme at the cost of reducing the number of bits available for video coding. Similarly, it is possible to increase the power of the channel codec assigned to the protection of the perceptually most important video bits at the cost of reducing the protection of the less important video bits. However, it is intractable to directly optimize the associated partitioning. Furthermore, it is possible to assign the video bits to a high number of bit protection classes; however, in most practical cases, it is sufficient to employ two or three protection classes [13].

The partitioning process is, in general, described with the aid of the percentage of bits assigned to the individual partitions. The number of different bit-sensitivity classes and the video bits assigned to them has to be decided on the basis of the visual importance differences of the various video bits, although the rigorous formal evaluation of these sensitivities is an arduous and time-consuming task. Hence, in a somewhat simplistic, but plausible approach, it is often argued that in a subjective sense, the visual importance of the various video parameters is related to the spatial frequency of the video features described by the parameters. This is also true in the case of interframe coding, when the objects move at a high velocity, since the combined effects of high-velocity, high-frequency video contents cannot be accurately resolved by the human eye due to its relatively low so-called fusion frequency.

The above observation is inherently exploited in subband [12] and Discrete Cosine Transform (DCT) [15,16] based coding, since typically a lower proportion of bits is assigned to the high-frequency video components than to their low-frequency counterparts. Hence, the partitioning of their bitstreams is relatively straightforward. The so-called inter-frame hybrid DCT coding technique has been adopted for most practical video codecs, including the existing video coding standards [7,8,9,10,11]. The partitioning of the associated video streams has also received considerable attention in recent years; e.g., in the context of Asynchronous Transfer Mode (ATM) networks for mitigating the effects of cell loss [17,18,19], and for providing SNR scalability—a term indicating that different quality video streams corresponding to different bitrates can be generated—as provisions in the MPEG-2 [11] and H.263 standards [9].

**Fig. 6:**
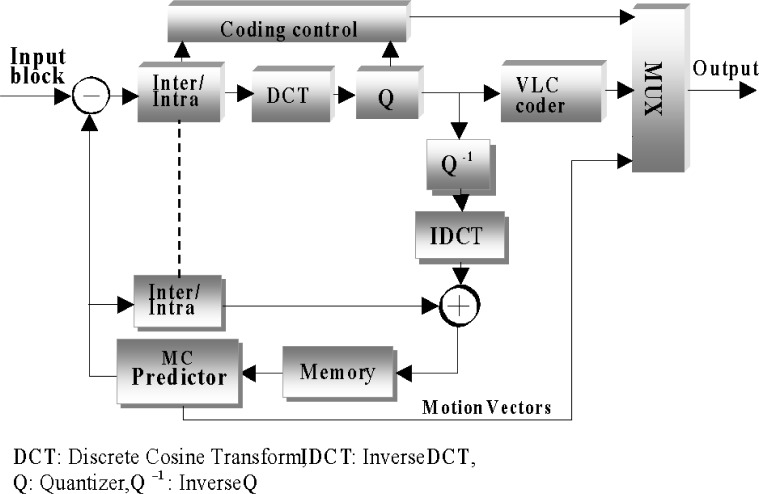
Block diagram of a hybrid DCT encoder.

The basic block diagram of an interframe hybrid DCT video encoder is depicted in Fig. 6. According to this approach, a video frame is first divided into non-overlapping blocks of 8 × 8 pixels, where each block is then DCT transformed, quantized (Q) and VLC coded. Except for the first video frame, which has to be intraframe coded (I-frame), the remaining frames may use a previously reconstructed frame known as the predicted or P-frame for motion prediction and compensation. At the cost of additional frame delays, both previous and future reconstructed frames may also be considered for motion prediction. This is known as bi-directional prediction, which has not been considered in our further elaborations.

For interframe prediction, a larger block of typically 16 × 16 pixels consisting of four neighboring 8 × 8 luminance DCT blocks—referred to as a macroblock (MB)—is used to perform block matching motion estimation and compensation. We note, furthermore, that a color MB also contains the so-called color difference components, which are processed at half the luminance resolution in vertical and horizontal directions. Since there are two color difference components, a MB can be viewed as though it was represented by six blocks. The estimated displacement motion vectors are multiplexed with the DCT coded data and transmitted as a part of the hierarchically ordered macroblock information.

The multiplexing structure of all existing video standards is generally based on a hierarchical, self-descriptive structure of the encoded parameters. For example, in the H.263 standard the video-coded information for each frame is arranged in four hierarchical layers. The top layer is the picture layer followed by a Group of Block (GOB) layer comprising a number of consecutive macroblocks, then the Macroblock layer, and finally a block layer. Each layer is furnished with some header information that may include synchronization bits such as picture start code, PSC, and GOB start code (for the two top layers), and that defines the nature of the information associated with each layer (e.g., inter/intra-type, quantization parameter, and motion vectors). If the header information of a specific video frame is lost during transmission, the decoder will have no indication as to how the frame, GOB, or MB has been coded. Therefore, any further data received will be undecodable, until the next PSC is recognized in the received bit pattern, e.g., by invoking correlation techniques.

As expected, the DCT coefficients associated with the particular video blocks are transmitted at the block layer and errors occurring in the DCT coefficients imply that the corresponding DCT coefficients are lost, since this information was variable length coded. If the transmission errors affected only higher frequency coefficients, the damage would be less catastrophic, since the more visually important low-frequency coefficients may have been recovered already. It is, however, important to protect the most error sensitive header information and as many lower frequency DCT coefficients as possible. The coding parameters hence have to be partitioned into a number of bit protection classes, in order to facilitate source-sensitivity matched error protection.

We should point out, however, that partitioning could result in a significant increase in the overall bitrate. This is caused by not only the additional partitioning overhead required for synchronization, but also by the coding inefficiencies that can arise from separating the DCT coefficients prior to variable length (VL) coding. For this reason, we argue that it is advantageous if partitioning can be accomplished in terms of the VL-coded DCT coefficients. In addition, we are concerned with developing a video stream partitioning scheme based on constant bitrate (CBR) transmission. We note, furthermore, that there are no fundamental requirements for the partitions to be of equal size. In fact, the partitions are often of differing sizes depending on the error protection strategy with respect to the channel conditions.

### 3.1 VLC-based Partitioning

As mentioned earlier, the separation of the quantised DCT coefficients into different protection classes can be arranged either before or after VL-coding. In the former case, i.e., when partitioning before VL-coding, which we refer to as fixed-zone partitioning, a given number of lower frequency DCT coefficients generated by the standard zigzag scanning process [16] is earmarked for transmission over a higher-integrity subchannel, which can be constituted, for example, by the higher-integrity transmission channel.

**Fig. 7:**
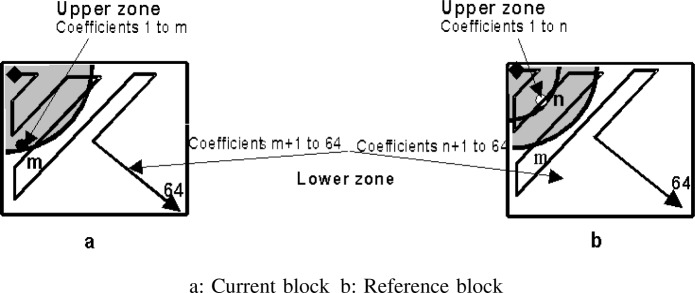
Zone difference in a VLC-based partitioning.

In our experiments we observed, however, that this arrangement could significantly increase bits per block, mainly due to the breakup of the run-level symbol near the DCT cutoff region [14]. Alternatively, if the block is partitioned after VLC coding, by selecting a fixed number of VLCs (i.e., VLC-based partitioning) no additional bits would be needed. The main concern with this approach, however, is that the number of DCT coefficients within the upper-zone may change from one block to the next. This is due the nature of run-level coding, where each VLC can represent a different number of DCT coefficients. Consequently, this affects the progression of noise at the receiver as the lower priority partition (second partition) is often expected to be lost. For a better clarification of the above argument, let us assume that the upper-zone, as shown in Fig. 7a, corresponds to the number of DCT coefficients *m* that is selected for the current coding block. In addition, suppose that, when the motion compensated block (reference block) was partitioned during the transmission of the previous frame, the number of its DCT coefficients for the first partition resulted in an “*n*” number of coefficients where *n* < *m*. The upper-zone difference between the two blocks is shown in Fig. 7b, where the darker shaded area represents the selected upper-zone region of the reference block. Recognizing that the coefficients within the zone difference belong to the second partition of the reference block, now consider the situation when the second partition had been corrupted by errors during the transmission of the previous frame. Under such a condition, the upper zone DCT representing the first partition of the current interframe block cannot be properly reconstructed due to the loss of DCT information within the zone difference. This would, consequently, cause a drift between the local and remote decoder, regardless of how well the first partition is protected. The visual impact of such distortion depends on the number as well as the magnitudes of the non-zero coefficients that fall within the zone difference, *m* − *n* [14]. However, the progression of such distortion and its visual effects does not appear to be of grave concern—as far as the intraframe reset can be accomplished in a reasonable period (see the results section).

### 3.2 Splitting Scheme

In order to develop a robust partitioning scheme, the splitting mechanism should take into consideration the instantaneous variations of the coded video. Since we are dealing here with CBR transmission, this is arranged in accordance with the measure of buffer fullness, as each partition is equipped with a separate buffer. The control management of these buffers is handled by the buffer control unit (BCU) [14]. Its function is to calculate and compare the occupancies between the two buffers. Further, it instructs the bitstream splitter to select one of the following options for the first partition: 0) picture and GOB headers, 1) all-headers, 2) all-headers + first VLC, 3) all-headers + first two VLCs. This information, which will be referred to as “cut-off value”, is represented by 0, 1, 2, and 3, respectively, and should be included in the header of the particular transmission layer on which it will be updated.

To avoid frequent buffer overflow/underflow we have observed that the cut-off value can be reasonably accomplished at the GOB level. More importantly, the cut-off value can be embedded into the Group Number (GN). The GN is a fixed length codeword of 5 bits. The bits are the binary representation of the GOB numbers in a frame. For a QCIF format (176 pixels by 144 lines per frame) there exist altogether nine GOBs. Since the first group of blocks do not require GOB information (as it is placed immediately after the picture information), three bits would be sufficient to transmit the group number (excluding the first GOB number). In our simulation model this has been arranged by sending the group number for the first two GOBs as “0” and the remainder as sequential numbers. With this arrangement, the two most significant bits of the 5 bit GN will be free and thus, can be utilized for transmitting the cut-off values. To preserve the integrity of the H.263 syntax, in the re-assembling process (i.e., pre-decoder) the group numbers will be put back to their original format before being decoded by the H.263 standard decoder. Under this arrangement, no extra bits will be added to the bitstream [14]. The only extra information would be for frame synchronization, such as picture start code (PSC), temporal reference code (TR), GOB start code (GBSC) and a GN codeword (3 bits only), which are added to the second bitstream at the beginning of each frame, including its GOBs. The decoder could use this to align the two bitstreams in order to make the second bitstream more robust to transmission errors.

**Fig. 8:**
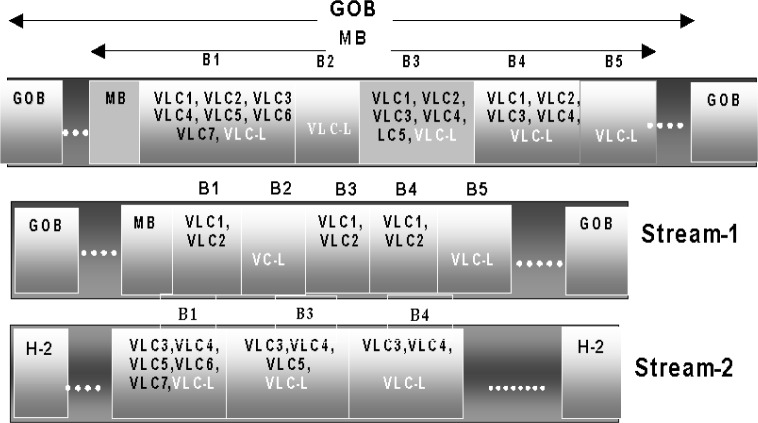
Two-layer partitioning for selection value of 2.

Next, we discuss the manner in which the two partitions are formed. For this purpose, let’s consider a scenario where the cut-off value indicates that at least two VLCs should be selected for the upcoming GOB. In this case, as shown in Fig. 8, the first partition begins with the GOB header followed by the MB header and the first two VLCs from each block in the transmitting order. This process will continue until the selection value is updated at the next GOB. The remaining VLCs are subsequently transferred to the second partition in the same order. It should be noted that the second bitstream does not carry any VLCs from the blocks whose last VLCs are included in the first partition or identified as zero blocks by the macroblock header (i.e., B2, B5, B6 in Fig. 8).

At the receiver, the two bitstreams are joined together to form the original H.263 bitstream via a pre-decoder unit. The pre-decoder’s initial task is to read the two most significant bits of the GN number to extract the cut-off value. From the cut-off value, the pre-decoder can determine from which of the two bitstreams it should obtain the macroblock information if the option-0 (split-header) has been identified. Otherwise, the pre-decoder can determine from the cut-off value how many VLCs it should read from the first bitstream. If the last VLC is not coded as a LAST-VLC (please note that a separate codeword is assigned to the last run-level symbol in a DCT block to signify the end-of-block), the remaining VLCs will then be read from the second bitstream until the last LAST-VLC is reached.

If an uncorrectable error has occurred in the second bitstream, the program is able to detect it but unable to pinpoint the exact location. Under such a condition, the second bitstream will be ignored for the entire GOB. However, to reconstruct the legal H.263 bitstream, every block within a GOB that does not contain a LAST-VLC (e.g., B1, B3, and B4 in Fig. 8) should be closed. This is done by replacing every codeword (e.g., second VLC according to Fig. 8) by its equivalent LAST-VLC codeword. If the cut-off value has identified an option-0 (split-header), all the blocks in the GOB will be considered uncoded. This is done by changing the COD flag. This situation normally occurs for P-frames only.

## 4. The Dual-Priority WCDMA Simulation Model

**Fig. 9:**
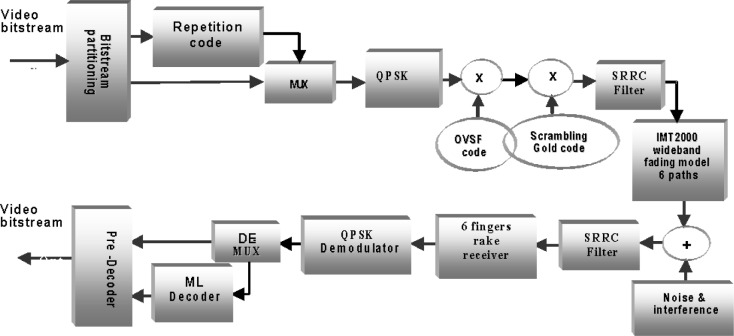
The system model for the multi-priority video transmission scheme over the 3Gpp downlink channel.

Figure 9 shows the system model for the multi-priority video transmission scheme over the 3GPP FDD downlink channel. The video signal, after being encoded, is partitioned into two separate bitstreams.

The first stream, containing the control and header information, is channel encoded using a simple repetition scheme where each bit is repeated three times. The second stream is passed through without encoding and multiplexed with the encoded stream. The multiplexed data is then sent over the 3GPP physical layer, where the resulting bits are first mapped onto a QPSK signal, spread using an OVSF code, and scrambled using a Gold code [5]. The resulting complex baseband signal is subsequently forwarded to a Square Root Raised Cosine (SRRC) filter. The roll-off factor of the SRRC filter is 0.22, as specified for 3GPP. The filtered signal is then sent through a wideband fading channel with 6 paths, in accordance with the IMT2000 specification (the parameters of the channel will be discussed later). Noise and interference are then added. The noise and interference have been modeled as Additive White Gauassian Noise (AWGN). The signal is then sent to the receiver SRRC filter, and then to a six finger Rake receiver where the signals on all the six paths are selected and combined coherently. The combined signal is then sent for QPSK demodulation.

Finally, the resulting bits are demultiplexed into two streams. The bits corresponding to the repetition encoded stream on the transmit side are sent to a majority logic (ML) decoder. The output of the ML decoder is sent for bit stream reassembly. The bits corresponding to the uncoded stream are directly passed through for bit stream reassembly. The reassembled video stream is then sent to the video source decoder to reconstruct the video signal.

### 4.1 Model Parameters

The simulation model for the transmission system was set up using the Signal Processing Worksystem (SPW)^3^. The parameters for the simulations are discussed below. The spreading factor was set to 64, which results in 80 bearer bits (40 symbols) in each slot. We used 16 of those bits or 8 symbols as pilot symbols, and the remaining 64 bits are used for data transmission. The pilot bits are placed at the beginning of each slot. The instantaneous pilot power was set 3 dB above the data. The chips are oversampled by a factor of 8. The SRRC filter with a roll-off factor of 0.22 was implemented with a complex Finite Impulse Response (FIR) filter with 128 taps. The channel impulse response for the vehicular IMT-2000 channel model is shown in Table 2. In our simulations, we assume ideal finger search for the Rake receiver. That is, we assume that each finger in the receiver has perfect synchronization with the corresponding path in the channel. We also assume perfect sub-chip synchronization (ideal phase locked loop). However, we used weighted multi-slot averaging (WMSA) for channel estimation in each finger. The channel estimation technique uses six consecutive slots for pilot estimation and then weighs the estimate across the six slots. The weighing for WSMA is shown in Table 3. The combining scheme is maximal ratio combining with finger selection. Only those fingers whose power is greater than 10 % of the maximum power are selected for combining. The majority logic decoder is a hard decision decoder. It chooses 1 if two or more 1’s are detected, and chooses 0 if 2 or more 0’s are detected. The simulations were run for a mobile speed of 60 km/h. For a carrier frequency of 2 GHz this translates to a Doppler frequency of 111.2 Hz.

Since a spreading factor of 64 was selected, the effective data rate used in our model was 96 kbit/s (only pilot channel consisting of eight symbols per slot was considered for DPCCH). For every one information bit (or 3 encoded bits) from stream 1, three uncoded bits of stream 2 are transmitted and the source coding rate is, therefore, 3/5 times the effective data rate. In other words, the data rate for the partitioned video signal is 57.6 kbit/s with a splitting fraction of *X* = 1/3 (see Fig.9). This would result in the following bitrates for each partitioned bitstream:

$$R_{\text{bistream}-1}=XR=\left(1/3\right)\;\left(57.6\right)=19.2\;\text{kbit/s}$$

$$R_{\text{bistream}-2}=\left(2/3\right)\;\left(57.6\right)=38.4\;\text{kbit/s}$$

where *R* is the video coded bitrate.

## 5. Results and Discussion

### 5.1 Video Partitioning Results

For the above splitting factor, the ITU-T H.263 based partitioning scheme discussed in Sec. 3, was then applied to split the first 405 frames of three sequences known as “Salesman”, “Claire”, and “Carphone” (note that all input sequences were generated at a frame rate of 30/s). As discussed, to embed the cut-off value in the GN codeword, these sequences conformed to the QCIF format. However, to comply with the low splitting fraction of *X* = 1/3 for the first partition, the coding frame rate was set at 10/s [14]. Furthermore, in these experiments, after every 135 interframe coded frames, the next frame was encoded as an intraframe mode (I-frame reset).

**Fig. 10:**
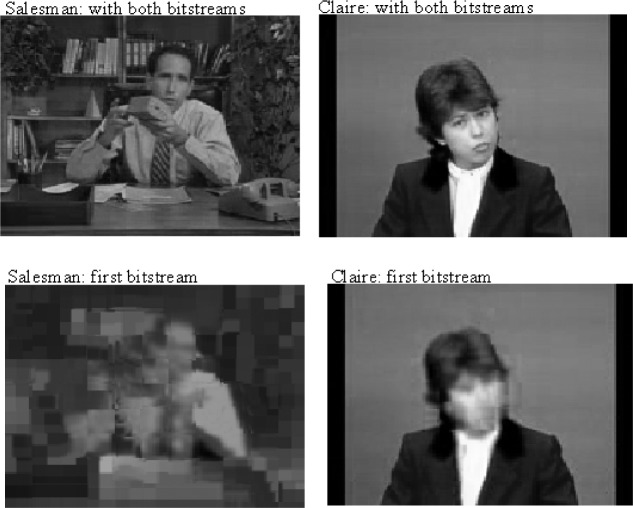
136th reconstructed frame of the Salesman and Claire sequences with and without the second bitstream.

Table 4 provides the video partitioning results with a splitting factor of *X* = 1/3, which includes detailed values of the coding and splitting parameters (excluding the GOB synchronization). In order to provide some subjective evaluations with regards to the distortion built up, we considered a situation where the entire second bitstream was corrupted by errors. Figure 10 depicts the 136th decoded frame of the “Salesman” and “Claire” sequences encoded at 10 frames/s and a bitrate of 57.6 kbit/s, with the splitting fraction of 1/3. For the sake of comparison, this figure also displays the reconstructed frames where the second bitstream is received error free.

### 5.2 Transmission

**Fig. 11:**
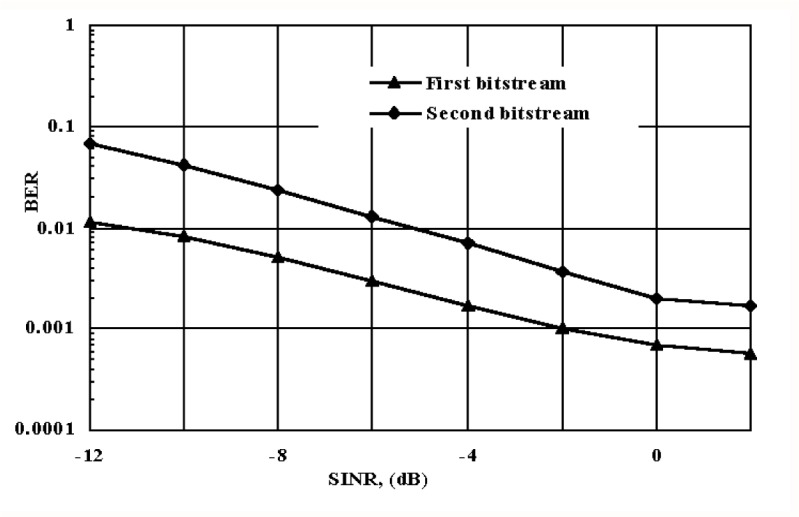
Bit-error-rate (BER) versus signal to interference noise ratio of the dual priority WCDMA based transmission system.

The final stage of our experiments was concerned with the transmission aspects of the partitioned video signal over WCDMA. The “Salesman” sequence with QCIF format and at the original frame rate of 30/s, was used as an input video in these experiments. The sequence contained 405 original frames and was encoded at a rate of 10/s. For a thorough evaluation of our transmission system, the sequence was repeated a hundred times to generate longer data. After the last frame was encoded (frame 136), the first frame of the repeated sequence was intraframe coded (I-frame). Thus, resulting in the I-frame reset period of 136 frames. Figure 11 shows the bit error rate (BER) performance verses the signal-to-interference noise ratio (SINR), when the partitioned video signal is applied to the system depicted in Fig. 9. As can be seen, using a simple repetition code can improve the SINR for the first bitstream by almost 4 dB.

In the process of reconstructing video, if errors were detected in the first bitstream, the entire data in the GOB (group of blocks) was replaced by the reconstructed GOB from the reference frame. This may, consequently, cause a drift between the local and remote decoder that tends to propagate until it reaches the next I frame. Such a drift would result in more severe distortion, particularly if errors occur on the I-frame data leading to the elimination of a portion, or even the entire, video data.

In the process of reconstructing video, if errors were detected in the first bitstream, the entire data in the GOB (group of blocks) was replaced by the reconstructed GOB from the reference frame. This may consequently cause a drift between the local and remote decoder that tends to propagate until it reaches the next I frame. Such a drift would result in more severe distortion, particularly if errors occur on the I-frame data leading to the elimination of a portion, or even the entire, video data.

If errors were detected in the second bitstream (please also refer to Sec. 3.2), its entire data within the corrupted GOB was eliminated. Consequently, all the blocks within the same GOB were reconstructed using the data received via the first bitstream. This was done by replacing every codeword by its equivalent LAST-VLC codeword. If the cut-off value was identified as a split-header, all the blocks in the GOB were considered uncoded (i.e., by changing the COD flag).

**Fig. 12:**
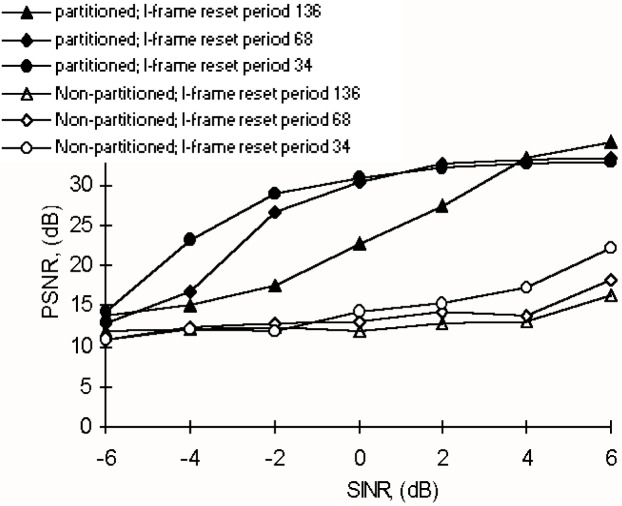
Average peak to peak signal to noise ratio (PSNR) of the reconstructed Salesman sequence verses SINR

Figure 12 shows the average peak-to-peak signal to noise ratio (PSNR) of the reconstructed frames using different I-frame reset periods ranging from 34-to-136 frames. As shown, with a default I-frame reset period of 136, which was imposed on the repeated sequence, the recovery of the video signal at lower SINR values is almost impossible. This is mainly due to excessive errors on the first bitstream, causing a loss of most error sensitive header information. Consequently, the distortion effect tends to propagate until reaching the next I-frame. For instance, as shown in Fig. 12, more frequent I-frame resets can enhance the PSNR of the reconstructed video. But this would be at the expense of reducing the video compression efficiency. Therefore, for the best performance, a careful balance between the level of FEC protection and the I-frame reset period would be needed with respect to the transmission channel conditions. Fortunately, in this case, the FEC overhead is added to protect a third of the input data. However, with better error protection it would be possible to employ a longer I-frame reset; thereby maintaining compression efficiency.

For the sake of comparison, the transmission of a non-partitioned H.263 coded bitstream was also carried out and the results are included in Fig. 12. For this case, the video was encoded at the higher bitrate of 102.4 kbit/s and transmitted via the same IMT-2000 channel. These results are presented using the same I frame reset periods ranging from 34 to 136 frames. As shown, except for higher SINR values, the recovery of the non-partitioned video is almost impossible, even at the lowest I-frame reset periods. For instance, let us compare the results of non-partitioned video coded at 96 kbit/s with the shortest I-frame reset period of 34, and that of the partitioned video coded at 57.6 kbit/s, but using the longest I-frame reset period of 136. We can observe that despite its lower bitrate, the partitioned video, as well as using a much larger I-frame reset period, is more resilient to errors when transmitted over IMT-2000 channels.

Finally, as the simulation results show, using this simple, hard-decision coding scheme results in a significant improvement in the recovery of the video signal compared to when no partitioning is applied. However, with more sophisticated FEC coding schemes, such as turbo codes and using better decoding schemes at the receiver, the performance can be expected to improve considerably. One obvious improvement would be to introduce an interleaver after the repetition encoder, and to use soft decision decoding to allow variable-bit-rate (VBR) video transmission together with the multi-priority scheme at the receiver. That is, instead of doing majority logic decoding, the outputs of the Rake receiver *r*_1_, *r*_2_, *r*_3_, corresponding to the transmitted and repeated symbol *s* (i.e., “*s*, *s*, *s*”) are combined to give *r* = *r*_1_ + *r*_2_ + *r*_3_, and *r* is then sent to the QPSK demodulator to obtain an estimate of the transmitted signal *s*.

Another effective FEC coding technique would be to use different FEC codes for each stream. For instance, stream 1 may be coded using a rate 1/2 turbo code, while stream 2 uses a rate 3/4 turbo code. Moreover, the 3GPP physical layer allows the change of data rate from frame-to-frame, in which case different rates and coding schemes may be used for each partitioned bitstream.

## 6. Conclusions

This paper presents a robust dual-priority video partitioning method suitable for twin-class unequal protected video transmission over IMT-2000 channels. The partitioning advocated is based on a separation of the Variable-length (VL) coded Discrete Cosine Transform (DCT) coefficients within each block. The method was shown to be suitable for constant bit rate (CBR) transmission, where the fraction of bits assigned to each of the two partitions can be adjusted according to the requirements of the unequal error protection scheme employed. The distribution of the VL-coded (VLC) information amongst the two partitions is performed adaptively. The results illustrate that if the second partition is corrupted by errors, the propagation of distortion does not cause a severe degradation to the reconstructed video. The partitioned video was then applied to one of the leading third generation cellular radio standard proposals, often referred to as the WCDMA system. A simple error correction coding scheme was employed to evaluate the performance of unequal error protection for transmission of ITU-T H.263 compressed video over IMT-2000 channels. The results were compared with non-partitioned video using more periodic I-frame encoding to reduce the propagation of distortion.

## Figures and Tables

**Table 1:** The bit rates, symbol rates, and spreading factors for WCDMA dedicated transport channels

Spreading factor (SF)	Symbol rate (thousand symbols per second)	bit rate kbit/s
512 (downlink only)	7.5	15
256	15	30
128	30	60
64	60	120
32	120	240
16	240	480
8	480	960
4	960	1920

**Table 2:** Channel impulse response for the IMT2000 Vehicular channel model

Delay (ns)	Average power (dB)
0	0
310	−1.0
710	−9.0
1090	−10.0
1730	−15.0
2510	−20.0
1090	10.0

**Table 3:** The weighting for WMSA

	Weight
1	0.3
2	0.8
3	1.0
4	1.0
5	0.8
6	0.3

**Table 4:** Results for Salesman, Claire, and Carphone sequences coded at 10 frames per second with a splitting fraction of 1/3

Video sequence	Salesman	Claire	Carphone
Header bits (kbit/s)	8.64	9.18	14.3
Coefficient bits (kbit/s)	48.812	48.48	43.2
Mean quantizer	5.62	4.51	10.28
Frames with split-header GOB’s (%)	28.67	17.6	91.6
Mean cut-off	1.1	1.24	0.39
Bitstream1 (kbit/s)	18.93	19.02	19.01
Bitstream2 (kbit/s)	38.53	38.65	38.54

## Footnotes

1 Chip is defined as a unit representing a bit after spreading every data symbol.

2 Figure 2 describes the base station baseband processing for a single traffic channel. The transmission for multiple channels may be combined differently based on the preferred implementation of the base station transmitter.

3 Signal Processing Work System and SPW are registered trademarks of Cadence Design Systems, Inc. Certain commercial equipment, instruments, or materials are identified in this paper to foster understanding. Such identification does not imply recommendation or endorsement by the National Institute of Standards and Technology, nor does it imply that the materials or equipment identified are necessarily the best available for the purpose.
